# Strong hybrid male incompatibilities impede the spread of a selfish chromosome between populations of a fly

**DOI:** 10.1002/evl3.55

**Published:** 2018-05-10

**Authors:** Rudi L. Verspoor, Jack M. L. Smith, Natasha L. M. Mannion, Gregory D. D. Hurst, Tom A. R. Price

**Affiliations:** ^1^ Institute of Integrative Biology University of Liverpool Liverpool L69 7ZB United Kingdom

**Keywords:** Compatibility, *Drosophila subobscura*, segregation‐distortion, selfish genetic elements, suppression, X chromosome meiotic drive

## Abstract

Meiotically driving sex chromosomes manipulate gametogenesis to increase their transmission at a cost to the rest of the genome. The intragenomic conflicts they produce have major impacts on the ecology and evolution of their host species. However, their ecological dynamics remain poorly understood. Simple population genetic models predict meiotic drivers will rapidly reach fixation in populations and spread across landscapes. In contrast, natural populations commonly show spatial variation in the frequency of drivers, with drive present in clines or mosaics across species ranges. For example, *Drosophila subobscura* harbors a sex ratio distorting drive chromosome (SR*s*) at 15–25% frequency in North Africa, present at less than 2% frequency in adjacent southern Spain, and absent in other European populations. Here, we investigate the forces preventing the spread of the driver northward. We show that SR*s* has remained at a constant frequency in North Africa, and failed to spread in Spain. We find strong evidence that spread is impeded by genetic incompatibility between SR*s* and Spanish autosomal backgrounds. When we cross SR*s* from North Africa onto Spanish genetic backgrounds we observe strong incompatibilities specific to hybrids bearing SR*s*. The incompatibilities increase in severity in F2 male hybrids, leading to almost complete infertility. We find no evidence supporting an alternative hypothesis, that there is resistance to drive in Spanish populations. We conclude that the source of the stepped frequency variation is genetic incompatibility between the SR*s* chromosome and the genetic backgrounds of the adjacent population, preventing SR*s* spreading northward. The low frequency of SR*s* in South Spain is consistent with recurrent gene flow across the Strait of Gibraltar combined with selection against the SR*s* element through genetic incompatibility. This demonstrates that incompatibilities between drive chromosomes and naïve populations can prevent the spread of drive between populations, at a continental scale.

Sex chromosome meiotic drivers are selfish genetic elements that spread by increasing their own transmission at a cost to the alternative sex chromosome (Burt and Trivers 2006). In heterogametic males, this means that more than 50% of functional sperm will bear the driving chromosome, allowing it to spread rapidly through populations. Sex chromosome meiotic drivers are a potent ecological and evolutionary force (Jaenike 2001; Lindholm et al. 2016), considered important in the evolution of sex ratios, mating rate (Price et al. 2008), and in causing coevolution through intragenomic conflict (Bastide et al. 2011).

Meiotic driving chromosomes can sweep rapidly through species ranges, and thus seem likely to prevent divergence and homogenize populations (Hamilton 1967). Alternatively, meiotic drive has been suggested to potentially create reproductive incompatibilities and hybrid sterility between populations (Frank 1991; Hurst and Pomiankowski 1991). These reproductive incompatibility models rely on intense intragenomic conflict and spatial variation in the direction and intensity of the conflict. The conflict can lead to selection on drivers for increasing transmission advantage and on rival chromosomes to evolve suppression of drive. The rest of the genome must also evolve to tolerate these rapid changes that are concentrated in spermatogenesis‐related genes (Meiklejohn and Tao 2010). This has the clear potential to create Dobzansky–Muller incompatibilities between populations that carry a driver and those that carry either no drivers or different ones (Frank 1991; Meiklejohn and Tao 2010; Patten 2018).

Thus, the observation that driving sex chromosomes can be found at stable, but spatially variable, frequencies is important, as this creates the context for the evolution of reproductive incompatibilities within a species (Lindholm et al. 2016). In the fly *Drosophila pseudoobscura*, a driving X chromosome referred to as “*Sex‐Ratio*” or “SR” shows clinal variation in North America, being present at low frequency in northern populations compared to southern ones (Sturtevant and Dobzhansky 1936; Price et al. 2014). The frequency of driving X chromosomes in *Drosophila simulans* is a geographical mosaic, with high frequency in some populations, medium in others, and absence in some (Bastide et al. 2011). In the house mouse populations, the autosomal driving t‐haplotype varies in frequency (Lenington et al. 1988). Past work on *Drosophila subobscura* indicates a stepped change in the frequency of the sex ratio distorting drive chromosome (henceforth referred to as “SR*s*”) between North Africa and Southern Europe, with SR*s* present at 15–25% frequency in North African samples, but at 0–2% in adjacent Southern European populations (Jungen 1967; Prevosti 1974; Hauschteckjungen 1990).

The causes of variation in drive frequency are known in some cases. For *D. pseudoobscura* and *D. neotestacea*, drive frequency is negatively associated with female remating rate, likely due to drive‐bearing males having substantially reduced success in sperm competition with nondrive males (Pinzone and Dyer 2013; Price et al. 2014), with evidence for a similar process occurring for the *t‐haplotype* in house mice (Manser et al. 2011; Sutter and Lindholm 2015). In *D. simulans* and *D. paramelanica* variation in resistance gene presence is important (Stalker 1961; Bastide et al. 2011). Abiotic conditions have also been proposed to influence drive frequency in *D. neotestacea* (Dyer 2012). In *D. subobscura*, however, the causes of differences in drive frequency across its range remain unclear.

Here, we examine the causes of variation in the frequency of the SR*s* chromosome in *D. subobscura*. SR*s* was first recovered and characterized as a case of sex chromosome meiotic drive in *D. subobscura* collected from Tunisia in the 1960s (Jungen 1967). Structurally, the SR*s* X chromosome is associated with a complex combination of four inversions that cover the majority of the X chromosome (Jungen 1967). The phenotypic strength of drive in males carrying the SR*s* chromosome is strong, and SRs/Y males produce broods that are 85–100% female (Jungen 1967; Hauschteckjungen 1990). Although population sex‐ratio skews have been associated with drive in other systems (Bryant et al. 1982), the impact of drive on population sex‐ratios has not been studied directly in *D. subobscura*.

In Tunisia where SR*s* was first described, it was found at frequencies of 15–25% (Jungen 1967, 1968). Later studies in southern Europe and Morocco between 1974 and 2002 revealed the SR*s* chromosome karyotype was present in Morocco at 0–25% frequency, in Southern Spain at 0–2% frequency, and was absent in Italian, French, and Northern Spanish populations (Prevosti 1974; Sole et al. 2002). Variation in female mating rate can be excluded as a cause of SR*s* frequency heterogeneity, as the species is monandrous in the populations tested for SR*s* (Verspoor et al. 2016). In addition, male fitness has been reported to be similar between SR*s* males and those not carrying drive (Hauschteckjungen et al. 1987) and no strong fitness costs have been demonstrated for homozygote and heterozygote females to date. Abiotic variables have been proposed to influence the frequency of the driving X chromosome in *D. neotestacea* (Dyer 2012); however, this remains to be explored in the SR*s* system. The reasons underlying the intermediate frequencies of SR*s* in North Africa, and the absence of SR*s* from Europe remain unclear.

Based on earlier work by Hauschteckjungen (1990), two alternate explanations for the spatial heterogeneity observed can be proposed. First, the SR*s* chromosome may be incompatible on genetic backgrounds outside North Africa. In support of this hypothesis, a cross where an SR*s* chromosome from Tunisia was placed onto the genetic background of a Swiss isoline resulted in infertile males (Hauschteckjungen 1990). If this incompatibility with SR*s* were to be similarly observed across diverse Southern Spanish genetic backgrounds, it could explain the stepped change in SR*s* frequency across the Straits of Gibraltar—SR*s* would be introduced by migration but then not spread. The second hypothesis to account for the failure of SR*s* to spread northwards is that there may be genetic resistance to the drive action in Southern European populations. Here, SR*s*/Y individuals are viable and fertile on a Southern European background, but drive does not occur and thus SR*s* does not spread.

In this article, we first assess the frequency of SR*s* in North African and Southern Spanish populations today to determine whether the stepped change in frequency is still present, and thus represents an equilibrium (rather than transient) condition. We then test the two hypotheses for explaining the rarity of SR*s* in Southern Spain. First, we examine whether the SR*s* chromosome is compatible with a Spanish genetic background. Second, we test whether there is any evidence of resistance to drive in Southern Spain that would inhibit spread. Our results suggest that the failure of SR*s* to establish in Southern Spain is caused by the presence of incompatibilities between SR*s* and the Spanish genetic background, with SR*s*/Y individuals having greatly reduced fitness in the presence of Spanish autosomes (while nondriving X chromosomes from North Africa are compatible). We hypothesize that it is intragenomic conflicts between SR*s* and autosomes that have driven this genomic incompatibility, which may therefore be a case of incipient conflictual speciation.

## Methods

### WILD FLY COLLECTIONS, FLY STOCKS AND THE FREQUENCY OF SRs FROM WILD POPULATIONS

We collected wild flies from four populations; Leeds, United Kingdom (53.86_N, 1.58_W) in May 2011; Tabarka, Tunisia (36.57_N, 8.45_E) and Punta Umbria, Spain (37.10_N, 6.57_W) in April 2013; and Amizmiz, Morocco (31.19_N, 8.25_W) in April 2016 (Fisher et al. 2013; Verspoor et al. 2015), using banana, yeast, and beer baits (Markow and O'Grady 2005). To assess SR*s* status of wild caught males, we mated them to a laboratory female to measure the sex‐ratio of the offspring they produced. One Tunisian male was also used to isolate the SR*s* driving chromosome to be used in hybrid crosses (Verspoor et al. 2016). A sex‐ratio of >85% females in a brood greater than 10 progeny was used to assign status of SR*s* to a male (Hauschteckjungen 1990).

Wild‐caught mated females were used to establish isofemale lines in the laboratory (David et al. 2005). Each isofemale line (or “isoline”) comprises the highly inbred descendants of a single wild‐mated female. By keeping multiple lines this process captures many wild genotypes from a population and minimizes adaptation to the laboratory. We maintained the isolines by mating full‐sibs to each other every generation. We inbred isolines for a minimum of eight generations before using them in experiments, and none of the isolines carried the SR*s* X chromosome. As each isoline represents a wild genotype, experiments using multiple isolines can test effects across genotypes. We also established “outbred” populations for experiments by freely mixing flies from all the isolines from a population (Table S1). For these outbred populations, we collected 50–100 virgin males and females from across the isolines. Each generation, five randomly chosen males and five females were housed together in each fly tube to mate and produce offspring, with 20–25 tubes per population per generation. These “outbred” populations were established for at least two generations before being used for experiments.

### INVESTIGATING HYBRID INCOMPATIBILITIES THAT COULD IMPEDE THE SPREAD OF SR*s*

#### Testing compatibilities of SR*s* versus non‐SRs chromosomes in native and naive populations

We searched for costs associated with the selfish SR*s* chromosome on naïve backgrounds by testing the fitness of three types of X chromosomes (a driving SR*s* X chromosome, a selection of nondriving X chromosomes from Tunisia, and a selection of nondriving X chromosomes from Spain) on different genetic backgrounds (either 100% from the population of origin, or a 50:50 mix of native and foreign backgrounds).

To this end, we crossed SR*s* homozygote females from Tunisia, and non‐SR*s* females from Tunisia and Spain to males from the focal outbred population. This produced experimental males with either their full native or 50% nonnative genetic background (Fig. S1a). For each of the six treatments, we paired 80 of these F1 hybrid males to a female to assess male fertility. These F1 males were generated from across 10 vials, with each vial containing five females from one of the three genetic origins (SR*s* homozygote, Spain population, Tunisia population) and five males from either the Spain or Tunisia “outbred” population. We counted each mating of an F1 experimental male as a replicate. The 80 females that we mated to the F1 experimental males from each treatment came from the Spanish and Tunisian outbred populations (40 per population), and female origin was included in the analysis. All flies used were seven days old to ensure sexual maturity (Holman et al. 2008), and paired for seven days to lay eggs. The number of offspring was counted and analyzed using analysis of variance (ANOVA) and Tukey's post hoc tests. Offspring sex ratio was used to confirm male X chromosome genotype (>85% female = SR*s*). All analyses were carried out in R 3.3.1 (R Development Team 2011).

#### Hybrid incompatibility of SRs males across three populations

We further tested whether hybrid incompatibility for SR*s* was confined to the adjacent Spanish population, or if distant populations that did not carry SR*s* were also affected. Here, we tested the strength of the hybrid incompatibility of SR*s* across different isolines from three populations (Tunisia, Spain, and the United Kingdom; Table S1). We produced experimental males by crossing SR*s* homozygote females to males from a selection of isolines (Tunisia—15 isolines; Spain—16 isolines; United Kingdom—eight isolines; Fig. S1b). This produced experimental SR*s* hybrid males with a 50% Tunisian SR*s* stock background and 50% Tunisian, U.K., or Spanish isoline genetic background. From each of the SR*s*/isoline F1 hybrids, we paired 40 males with a virgin female from an outcrossed Tunisian population and recorded the number of offspring produced as described in the previous section. To avoid pseudoreplication, we calculated a mean number of offspring and sex‐ratio for each isoline cross. We used ANOVAs to test for an effect of population of origin on the number of offspring produced and the strength of drive.

#### Fitness of the SRs chromosome in an F2 hybrid genetic background

We then investigated whether fitness costs of SR*s* chromosomes were higher on increasingly nonnative genetic backgrounds. To this end, we tested the fitness of SR*s* when it had been introgressed for two generations into a Spanish background.

We produced F1 heterozygote females by crossing homozygote SR*s* females to males from three Spanish isolines (Pum O3, N4, and S10). To produce the F2 experimental males, we then backcrossed F1 heterozygote X_SR_ *_s_*/X females to males from the same three isolines. The resulting male offspring now carried either an SR*s* or a Spanish nondriving X chromosome with a ∼25% Tunisia/75% Spain genetic background. We mated these focal males to an outbred Spanish female, and recorded the number of offspring produced. Focal males that produced fewer than five offspring could not be reliably assigned by offspring sex ratio, and so their X chromosome type was confirmed by sequencing the *G6P* gene region in both directions and using gel electrophoresis or SNPs to identify the chromosome of origin (see SOM for details). The number of offspring produced could not be normalized and was analyzed using a Wilcoxon rank test. The number of offspring produced by F1 hybrids and F2 hybrids carrying SR*s* was also compared using a Wilcoxon rank test.

#### Testing for rescue of SRs phenotype by backcrossing to the Tunisian genetic background

As an additional validation of the role of genomic background in male fertility, we tested whether SR*s* fertility could be rescued by increasing the proportion of the background that was Tunisian. We crossed half of the few F3 females generated above to a random male from an outbred Tunisian population, and half to an outbred Spanish population. As the focal females were heterozygotes, carrying one SR*s* chromosome, half of their sons would be expected to carry SR*s*. Male offspring of each focal female were mated as above. Males were subsequently assigned to three phenotype categories: SR*s* if the sex ratio of their offspring was >85% female, nondriving if the sex ratio was 50:50, and unknown if they produced five or fewer offspring. If Tunisian autosomes do rescue SR*s*, then the SR*s* phenotype would only appear in the backcross to Tunisian males, as SRs would remain sterile in the Spanish background.

#### Fitness of Tunisian SRs background in Moroccan genetic backgrounds

Incompatibilities could potentially be due to geographic isolation, rather than SR*s* presence. As a follow‐up experiment, we tested for SR*s*‐based incompatibilities between populations in North Africa that carried similar SR*s*, but were geographically distant. We crossed the homozygote Tunisian SR*s* females to males from 11 isolines from Amizmiz, Morocco (Table S1) to produce F1 50/50 Tunisia/Morocco hybrid males. We used SR*s* on a 100% Tunisian background as a control for the strength of drive and offspring production. For each isoline and the Tunisian control, we mated up to 25 F1 males to a virgin outbred Tunisian female and offspring number and sex ratio recorded. Mean offspring production for each line was analyzed using an ANOVA.

### TESTING FOR EVIDENCE FOR SUPPRESSION OF THE SR*s* DRIVE PHENOTYPE

#### Sex‐ratio distortion of SR*s* males across multiple populations

If the rest of the genome has evolved resistance against the sex‐ratio distortion of drive, we expect genetic variation in the sex ratios produced by males carrying SR*s*. If resistance prevents spread, we expect increased son production by SR*s* when on a Spanish genetic background.

We tested whether the proportion of sons produced by SR*s* differed when it was in hybrid genetic backgrounds between different populations. The data on sex ratio was generated from the same crosses as outlined in the Section “Hybrid incompatibility of SRs males across three populations.” Proportion of female offspring was arcsine transformed before analysis. Only crosses that produced more than five offspring were included in the analysis (see Table S1 for number of individuals per line). Isoline was the unit of replication, with a mean sex ratio calculated for each line, and we compared populations using ANOVA and Tukey's post hoc tests.

#### Fertility and Y‐chromosome status of sons of SRs males

In some systems, sons of meiotic drive males are actually infertile pseudomales, and so do not represent true suppression of drive (Cobbs 1992). To check that the few sons produced by Tunisian SR*s* fathers were not pseudomales, a subset of sons were tested for fertility by pairing them to two random outbred Tunisian females. After mating the sons as described in Section “Wild fly collections, fly stocks and the frequency of SR*s* from wild populations” of the Methods, vials were checked for larval action to confirm male fertility. The sons were then assayed for the presence of a Y‐chromosome using the *kl2* marker (Herrig et al. 2014). The presence of a Y‐chromosome was confirmed using gel electrophoresis with a positive and negative control.

## Results

### SR*s* FREQUENCY REMAINS CONSISTENT IN NORTH AFRICA AND SPAIN

**Figure 1:**
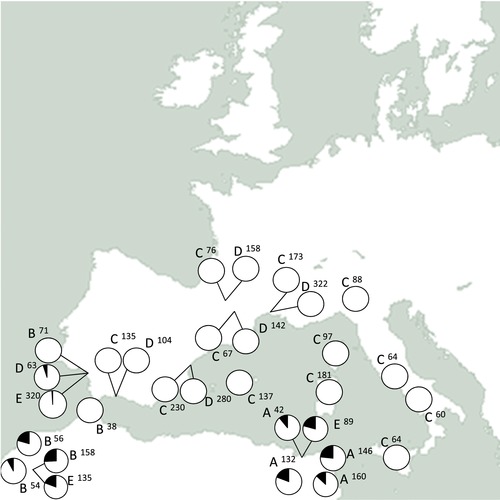
Map of driving and nondriving X chromosomes across Southern Europe and North Africa. Pie charts show the proportion of SR*s* (in black) and nondriving (white) X chromosomes. The numbers represent the years and sources of the collections (A—1968 [Jungen 1968], B—1974 [Prevosti 1974], C—1984 [Prevosti et al. 1984], D—2002 [Sole et al. 2002], E—2013–15 collections Table S2). Numbers next to the letters represent the number of X chromosomes sampled.

Wild males collected from three populations were screened for the SR*s* phenotype to compare current frequencies to previous collections. We found evidence for moderate levels of the SR*s* phenotype in males screened from Morocco (18%, *n* = 135) and Tunisia (12%, *n* = 89) (Fig. 1, Table S2). We found evidence for the SR*s* phenotype at very low frequency in south Spain, where it was only detected in only two males (∼0.5%, *n* = 320) (Fig. 1, Table S2).

### STRONG INCOMPATIBILITIES REDUCE SR*s* FITNESS ON NAÏVE GENETIC BACKGROUNDS

#### The compatibility of SRs versus non‐SR*s* chromosomes in native and naive populations

The number of offspring produced by the six types of hybrid male (three types of X chromosome: SR*s*, nondriving Tunisian, and nondriving Spanish; two genetic backgrounds: Spanish or Tunisian) was examined to test for fitness in hybrids. The origin of the female the hybrid male was mated to had no impact on offspring production or sex ratio (ANOVA: *F*_1,418_ = 0.474, *P* = 0.492), so this factor was removed from onward analyses.

**Figure 2:**
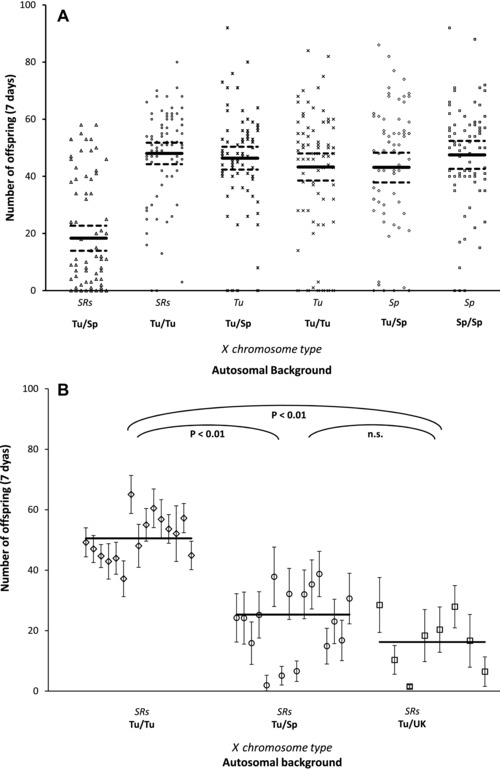
(A) The number of offspring produced by three different categories of X chromosome (SRs, nondriving Tunisian—Tu, nondriving Spanish—Sp) on different autosomal backgrounds (100% native autosomes or 50% foreign autosomes). Reduced fitness only occurs when SRs chromosomes occur in a hybrid background. Solid and dashed lined show the means and two SEM, respectively. (B) Mean number of offspring produced by SR*s* males with different genetic backgrounds, showing low fitness on hybrid backgrounds. Each point indicates the mean and 95% confidence intervals for a single isoline. Main lines show population means.

Across the six types of hybrid males, we observed a highly significant interaction between X chromosome type and genetic background (Fig. 2A; ANOVA: *F*_2,419_ = 30.64, *P* < 0.001). Males that carried a nondriving X chromosome showed equally high levels of fitness, irrespective of whether they were on a Spanish, Tunisian, or mixed genetic background (Tukey's post hoc test: *P* > 0.861 in all comparisons; Table S3). The fitness of SR*s* males that had a Tunisian genetic background equaled the high fitness of nondriving males (Tukey's post hoc test: *P* > 0.875; Table S3). In contrast, males that carried SR*s* on a mixed Spanish/Tunisian genetic background produced fewer than half the offspring of all other male types (Tukey's post hoc test: *P* < 0.001 in all comparisons; Table S3). The offspring production of the SR*s* hybrids on a mixed Spanish and Tunisian background appeared to be bimodally distributed, confirmed by Hartigan's dip test (*n* = 78, *P* = 0.0348).

#### Hybrid incompatibility of SRs males across three populations

To examine whether the F1 hybrid incompatibility was restricted to the adjacent southern Spanish population or was present in populations not exposed to SR*s*, we crossed SR*s* into a number of isofemale lines from the native Tunisian population, the neighboring Spanish population, and a distant U.K. population. There was equally strong evidence of incompatibility when the SR*s* chromosome is expressed on F1 hybrid backgrounds from both neighboring (Spain) and distant (United Kingdom) populations (Fig. 2B; ANOVA: *F*_2,36 = _43.91, *P* < 0.001; Table S4).

#### The fitness of the SRs chromosome in an F2 hybrid genetic background

**Figure 3:**
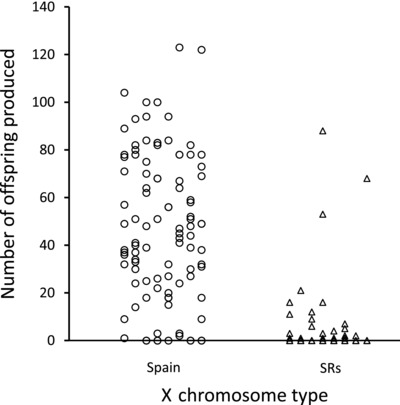
Scatterplot showing the number of offspring produced in seven days for males, which were introgressed into the Spanish population for two generations, carrying the Spanish X chromosome or the SRs chromosome. The X chromosome identity of males was confirmed using the SNP variation in the G6P locus (Supporting Information 1).

The ability of SR*s* to introgress into the Spanish population was tested further by exposing the SR*s* chromosome to an increasingly Spanish genetic background in an F2 backcross. This cross, which creates males that carry SR*s*, but carry 75% Spanish autosomal genes, resulted in almost complete infertility of SR*s* males. Over 90% of these males produced fewer than five offspring, compared to Spanish X chromosomes, which show normal offspring production (Fig. 3; Wilcoxon rank: *n* = 176, *W* = 6958, *P* < 0.001). We also tested if the fitness cost was more severe in F2 hybrids than F1 hybrids from Figure 1A. The F2 hybrids produced significantly lower number of offspring than F1 hybrids (Wilcoxon rank: *n* = 157, *W* = 4690, *P* < 0.001), indicating increasing fitness loss of SR*s* with increasing genomic content from southern Spain.

#### Testing for rescue of SRs phenotype by backcrossing to the Tunisian genetic background

We tested if reintroducing Tunisian genetic material could rescue the fertility of the SR*s* chromosome by backcrossing a small number of F3 offspring to Tunisian males. The resulting F4 SR*s* sons from crosses that reintroduced Tunisian genetic material had restored fertility (Table S5).

#### The fitness of Tunisian SR*s* background in Moroccan genetic backgrounds

**Figure 4:**
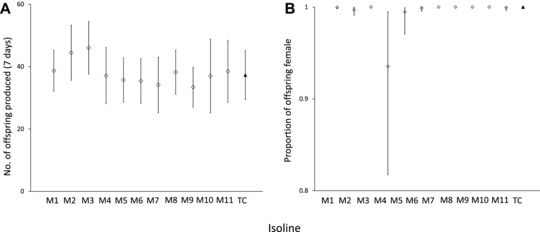
(A) Total offspring and (B) offspring sex ratios produced by hybrid Moroccan/Tunisian males carrying the Tunisian SR*s* (diamonds) and pure Tunisian control males carrying SR*s* (solid triangle). Points indicate the mean, whereas error bars indicate 95% confidence intervals.

Reproductive incompatibilities may be due to spatial isolation itself, rather than spatially varying presence of SR*s*. To test this, the SR*s* chromosome from Tunisia was crossed into a range of Moroccan isolines, which are geographically more distant than those of Spain, and the fitness of SR*s*/Y hybrid males tested. There was no evidence of hybrid incompatibility; hybrid crosses between Moroccan isolines and the Tunisian driver did not differ significantly in the number of offspring they produced when compared to a fully Tunisian background (ANOVA: *F*_11,245_, *P* = 0.318; Fig. 4). There were no significant differences between any of the isolines using Tukey's post hoc tests (all *P* > 0.3).

### NO EVIDENCE FOR SUPPRESSION PREVENTING THE SPREAD OF DRIVE

#### Sex‐ratio distortion of SRs males across multiple populations

**Figure 5:**
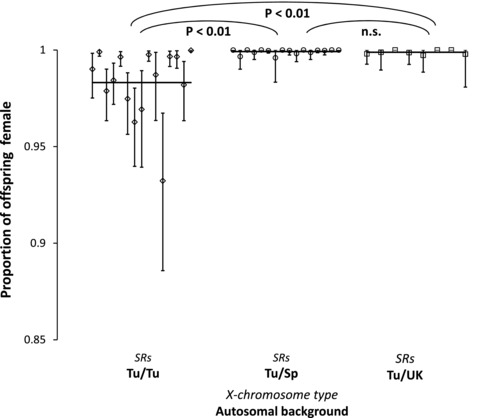
Offspring sex ratio of SR*s* males on native and Spanish/Tunisian and United Kingdom/Tunisian hybrid backgrounds. Each point indicates the mean and 95% confidence intervals for a single isoline. Main lines show population means.

One potential cause of the evolution of incompatibility between populations is the evolution of suppression of drive in the native population, which then selects for stronger drive, potentially producing “overdrive” on naïve backgrounds. This evolutionary process would be supported by the observation of stronger drive on naïve backgrounds compared to native, whereas stronger drive in native backgrounds would indicate suppression of SR*s* in Spain, potentially acting as a barrier to SRs spread. We therefore compared the strength of drive in three different populations, Tunisia, Spain, and the United Kingdom. We observed that drive (as measured by proportion of daughters produced) is stronger in hybrids with partial Spanish and U.K. genetic backgrounds than in the Tunisian background (ANOVA: *F*_2,36 = _17.71, *P* < 0.001; Fig. 5) due to weak suppression of SR*s* in Tunisia (Tukey's post hoc test: *P* < 0.0.01 in both comparisons; Table S6). Figure 5 also highlights that there are differences in the strength of drive between isolines from Tunisia. In contrast, the strength of drive appears to be consistently strong in all genetic backgrounds from Spain and the United Kingdom.

We tested whether the sons produced by SR*s*/Y individuals in Tunisia were fertile, and represented true suppression (rather than pseudomales associated with nondisjunction, as seen in other systems) (Cobbs 1992). We examined a random selection of the male offspring from lines showing weak suppression in order to establish if those male offspring were fertile and carried an X chromosome. We found that all but one of these males was found to be both fertile and to carry a Y‐chromosome, demonstrating that there is true suppression in North Africa (Fig. S2).

## Discussion

Meiotic drivers are potent evolutionary forces, but their ecological dynamics remain poorly understood (Lindholm et al. 2016). In this study, we examined the causes of the difference of frequency in SR*s*, a driving X chromosome, in the monandrous fruit fly *Drosophila subobscura*. Our field collections of *D. subobscura* from three populations (Tunisia, Morocco, and southern Spain) confirm that the SR*s* phenotype is still present in all three locations (Fig. 1). Frequencies of SR*s* were similar to previous samplings from Tunisia and Morocco (Jungen 1967; Prevosti 1974; Hauschteckjungen 1990). However, in southern Spain, we found the drive phenotype at slightly lower frequencies than previous reports (Sole et al. 2002). These results are consistent with the polymorphism of SR*s* being roughly stable over the last 50 years in North Africa and consistently low over that last 20 years in southern Spain.

What prevents SR*s* from increasing in frequency in Spanish populations? Our principle finding is that the lack of introgression of SR*s* into southern Spain is associated with severe genetic incompatibilities between SR*s* and Spanish genetic backgrounds. Motivated by previous findings of hybrid failure between SR*s* and a Swiss isogenic lineage (Hauschteckjungen 1990), we tested whether hybrid failure commonly occurred for SR*s* on the genetic background of the adjacent population. We observed strong SR*s* hybrid incompatibilities when SR*s* is found in Spanish genetic backgrounds. This hybrid incompatibility is not found for nondriving X chromosomes from Tunisia. This drive‐specific incompatibility thus represents a powerful impediment to the spread of SR*s* in Europe. In contrast, we find no evidence for genetic suppression in Spanish populations that might prevent the spread of SR*s* into Spain, and indeed drive was stronger in this population than on the native genetic background. These results demonstrate interpopulation incompatibilities represent a potent mechanism that can prevent the spread of a driving chromosome, a result congruent with evidence that spore killers can be restricted to particular populations of fungi (Turner 2001). In this case, strong hybrid incompatibilities specific to a driving chromosome are blocking it from spreading into south Spain.

What is causing the evolution of these incompatibilities? This type of X chromosome driver could cause rapid evolution by a number of means (Meiklejohn and Tao 2010). Population specific coevolution caused by genetic conflict between drivers and suppressors is one plausible explanation (Johnson 2010; Crespi and Nosil 2013). In naïve genetic backgrounds where this suppression is absent, the drive phenotype might express at a higher level, becoming toxic even to sperm carrying the driving X. Genetic suppression of selfish driving chromosomes has been observed in a number of other systems (e.g., Stalker 1961; Bastide et al. 2011) and we do find evidence of very weak suppression in North Africa. However, the role of suppressor evolution and overdrive awaits a more detailed account of the genetic factors associated with the production of sons from SR*s* males. An alternative explanation is that SR*s* in North Africa damaged the fertility of males that carried it, causing rapid evolution in genes involved in spermatogenesis to reduce these costs. In naïve populations without these compensatory alleles, SR*s* might then suffer severe fertility costs (Meiklejohn and Tao 2010). Currently we cannot determine whether either of these mechanisms created the incompatibilities.

The observations to date represent interactions between the SR*s* chromosome as a whole and Spanish autosomes: they do not causally link the locus/loci that cause SR*s* drive itself to the incompatibility. It is also feasible there are other loci within the complex architecture of the SR*s* chromosome contributing to incompatibilities. These loci could even be cryptic drive systems themselves. Multiple driving loci have been found in the *D. simulans*–*D. mauritiana* system, some of which are cryptic (Meiklejohn et al. 2018). Future work will need to examine the mechanism underlying this incompatibility in depth and how it relates to the SR*s* drive sperm killing phenotype in order to demonstrate the role of the driver (as opposed to linked variants) in causing incompatibility. If the driver is shown to be causally associated with incompatibility, it will represent strong evidence in support of the hypothesis that drive/autosome coevolution may cause the primary stages of reproductive isolation (McDermott and Noor 2012).

The role of selfish X chromosomes in speciation is receiving renewed attention since it was first proposed (Frank 1991; Hurst and Pomiankowski 1991) as evidence that supports it has accumulated over the last 20 years (Patten 2018). Should selfish X chromosomes always generate population‐specific coevolution and incompatibilities, or will they sweep through population after population, eventually covering the species range? Recent evidence suggests a driving X locus may have crossed a species boundary between *D. mauritiana* and *D. simulans*, resulting in the homogenization of genetic ancestry between two closely related species (Meiklejohn et al. 2018). A degree of population isolation, or a biotic or abiotic variable preventing the spread of the selfish X chromosome is likely to be required to stop the chromosome from immediately sweeping through the species range. In *D. subobscura*, this barrier could have been from the isolation of North African populations of *D. subobscura* during recent periodic glaciation events. Understanding when and how selfish X chromosomes generate incompatibilities remains an important question.

Could incipient hybrid incompatibilities be present in other drive systems? Both between subspecies and sister species there is already strong evidence for drive loci being associated with incompatibilities (Tao et al. 2001; Phadnis and Orr 2009; McDermott and Noor 2012). Incompatibilities created either coevolution between suppressors and drivers or more broadly with spermatogenesis‐specific genes could plausibly exist in other systems. In many systems, suppressors of drivers occur across populations (see Jaenike 2001 for review). X chromosomes may also become graveyards for inactive drive systems that are only revealed in interpopulation and interspecies crosses. Equally, if these incompatibilities are caused by linked variants that are locked up in large driving inversions, large inversions are not unique to the SR*s* system. Driving chromosomes often have large inversions, which reduce recombination and creates linkage across large areas regions (e.g., Babcock and Anderson 1996; Dyer et al. 2007).

The system allows us a unique opportunity to gain insights into the early origins of incompatibility associated with meiotic drive. It is likely the SR*s* system experienced a degree of historic subdivision between populations. Is some subdivision and barrier to gene flow necessary? If so, other systems where species have restricted gene flow due to climate history or geographic isolation may be candidates for the same process. However, in the case of SR*s* it is still unknown if this incompatibility evolved in the form of the accumulation of minor incompatibilities or as one or two single large contributing loci. Determining the number and age of the loci that are contributing to this incompatibility would prove informative to understanding how these incompatibilities initially begin to form. Excitingly, our observation is of incipient incompatibilities in process. For this reason, the system provides a window to understanding the formation of early incompatibilities between populations, and could help answer some of these fundamental questions of process.

Associate Editor: S. Wright

## Supporting information

Supporting information

**Figure S1**. The layout of the crossing schematics for (a) Experiment 1 comparing the fitness of the SRs X chromosome and nondriving X chromosomes from Tunisia and Spain on native and hybrid populations genetic backgrounds; (b) Experiments 2 and 4 comparing the fitness costs of SRs and the levels of suppression of SRs in multiple isofemale lines across three populations.

**Figure S2**. Gel electrophoresis image showing the amplification of the kl2 gene from the *Drosophila subobscura* Y chromosome.

**Table S1**. List of the isolines collected from the four locations that were used in the experiments.

**Table S2**. The proportion of female offspring produced from wild males caught in Tunisia and Spain collected in 2013.

**Table S3**. Tukey's post hoc tests on the differences in offspring produced by three types of X chromosome (Driving SR*s*—“SR*s”*, nondriving Tunisian—“Tun” and nondriving Spanish—“Spa”) on two different population genetic backgrounds (100% their own native background—Nat or 50%/50% their own background and that of a different population—Hyb).

**Table S4**. Differences in the number of offspring produced by SR*s* males when introgressed onto 39 isolines across three populations (Spain, *n* = 16; Tunisia, *n* = 15; United Kingdom, *n* = 8).

**Table S5**. The offspring production, offspring sex ratio, and the X chromosome status of males produced by backcrossing hybrid females carrying one SR*s* and one Spanish X chromosome to either a Tunisian or a Spanish male.

**Table S6**. Differences between three populations in the proportion of offspring that are female when an SR*s* male was introgressed onto an isoline from that population.
