# Supplementary material for: Strong hybrid male incompatibilities impede the spread of a selfish chromosome between populations of a fly
Source: Evol Lett. 2018 May 10;2(3):169–79. doi: 10.1002/evl3.55 (PMC6121854; doi:10.1002/evl3.55)
Supplement: Supplementary file 1 — Supporting information [file EVL3-2-169-s001.docx]

Supplementary Information for Verspoor et al 2018 Evolution Letters: Strong hybrid male incompatibilities impede the spread of a selfish chromosome between populations of a fly.

Supplementary Figure 1 showing the layout of the crossing schematics for a) Experiment 1 comparing the fitness of the SRs X-chromosome and non-driving X-chromosomes from Tunisia and Spain on native and hybrid populations genetic backgrounds b) Experiment 2 and 4 comparing the fitness costs of SRs and the levels of suppression of SRs in multiple isofemale lines across three populations. Colours indicate different genetic backgrounds from different populations. Checked pattern indicates the SR*s* X-chromosome is present in the cross.

Supplementary Figure 2. Gel electrophoresis image showing the amplification of the kl2 gene from the *Drosophila subobscura* Y-chromosome. PCR conditions were an initial 3min denaturing step, followed by 35 cycles of 94 for 30secs, 60 for 30 secs, 72 for 30secs, with a final elongation period of 10mins at 72. PCR products were determined using gel electrophoresis on a 1.5% agarose gel with 3μL Midori green per 100mL of TAE buffer. This image confirms that the few male offspring produced from SR*s* carrying males are carrying a Y-chromosome. All the males which carried a Y-chromosome were also found to be able to produce offspring when mated to a virgin female.

Supplementary information 1 - G6P locus

The G6P locus, located on the X-chromosome (A chromosome) was used to differentiate SR*s*, from Spanish X-chromosomes. The forward and reverse primers used can be seen below (Forward primer – ATCATACCGCTCTGGATCTCAT, Reverse primer – GTGGAGCTGAGGATCTTGTTG). The reaction profile was an initial 3min denaturing step at 95°C, followed by 35 cycles of 95°C for 30secs, 60°C for 30 secs, 72°C for 30secs, with a final elongation period of 10mins at 72°C. PCR products were determined using gel electrophoresis on a 2.5% agarose gel with 3μL Midori green per 100mL of TAE buffer. For one of the Spanish X-chromosomes, there was no amplification so SRs was scored based on the presence of a PCR product. For the two remaining X-chromosome types Sanger sequencing was used to identify the X-chromosome by SNP variation. PCR products were cleaned using antarctic phosphatase and exonuclease 1, with an incubation of 45mins at 37°C followed by 15mintes at 80°C. Sequencing products were amplified using BigDye3.1 protocol with a sequencing program of 35 cycles of 96°C for 10secs, 50°C for 5secs, 60°C for 4mins. Sequencing was precipitated using 3M sodium acetate and cleaned with 70% ethanol. 10ul of Hi-Di formamide was then added and sequencing was carried out on and ABI3500xL genetic analyser. SNPs in the region were called using the software Geneious version 7.1.3.

Supplementary Table 1 lists the isolines collected from the 4 locations that were used in the experiments. Dates of the collections are also listed next to the locations. Numbers in superscript represent the number of pairs that produced more than 5 offspring and so could be used for estimating suppression for different isolines

| Tunisia (2013) | Spain (2013) | UK (2011) | Morocco (2015) |
| --- | --- | --- | --- |
| TabA31 ^35^ | PumN4 ^18^ | DS4 ^19^ | AmzC4 ^24^ |
| TabA33 ^39^ | PumN16 ^2^ | DS13 ^18^ | AmzC5 ^23^ |
| TabB8 ^37^ | PumN24 ^29^ | DS16 ^20^ | AmzC1 ^23^ |
| TabB11 ^38^ | PumO3 ^28^ | DS20 ^21^ | AmzB3 ^22^ |
| TabB13 ^36^ | PumO11 ^26^ | DS23 ^12^ | AmzB15 ^23^ |
| TabB21 ^32^ | PumO15 ^31^ | DS24 ^11^ | AmzC6 ^23^ |
| TabB29 ^34^ | PumP4 ^25^ | DS33 ^22^ | AmzC17 ^19^ |
| TabC2 ^32^ | PumP7 ^27^ | DS35 ^10^ | AmzB13 ^19^ |
| TabC9 ^30^ | PumR2 ^14^ |  | AmzC2 ^19^ |
| TabC18 ^34^ | PumR4 ^20^ |  | AmzB5 ^13^ |
| TabC26 ^34^ | PumR7 ^27^ |  | AmzC21 ^9^ |
| TabC28 ^34^ | PumR8 ^10^ |  |  |
| TabC29 ^23^ | PumS4 ^29^ |  |  |
| TabC39 ^13^ | PumS8 ^22^ |  |  |
| TabE4 ^36^ | PumS10 ^10^ |  |  |
|  | PumS12 ^24^ |  |  |

Supplementary Table 2 shows the proportion of female offspring produced from wild males caught in Tunisia and Spain collected in 2013. Males were each crossed to a 7 day old virgin female from their same population of origin. Males were conservatively classed as the SR*s* phenotype if they produced >85% female broods (Hauschteckjungen, 1990).

| Proportion of offspring female | Tunisia (n= 146) | Spain (n = 320) | Morocco (n=135) |
| --- | --- | --- | --- |
| 0.3-0.4 | 1 | 9 | 8 |
| 0.4-0.5 | 32 | 86 | 22 |
| 0.5-0.6 | 83 | 175 | 50 |
| 0.6-0.7 | 22 | 41 | 21 |
| 0.7-0.8 | 3 | 7 | 8 |
| 0.8-0.9 | 3 | 0 | 3 |
| 0.9-1.0 | 12 | 2 | 23 |

Supplementary Table 3 showing Tukey’s post hoc tests on the differences in offspring produced by three types of X-chromosome (Driving SR*s* - "SR*s*", non-driving Tunisian – "Tun" and non-driving Spanish – "Spa") on two different population genetic backgrounds (100% their own native background – Nat or 50%/50% their own background and that of a different population – Hyb). The replicates for each category are as follows (SR*s*:Hyb n=61, SR*s*:Nat n=75, Spa:Hyb n=77, Spa:Nat n=70, Tun:Hyb n=73, Tun:Nat n=71)

| Tukey’s HSD tests SRs specific incompatibility (fig 2a) | | | | |
| --- | --- | --- | --- | --- |
|  | Difference | lower CI | Upper CI | P adjusted |
| SRs:Hyb - Spa:Hyb | 24.808 | 32.969 | 16.646 | < 0.001 |
| Tun:Hyb - Spa:Hyb | 0.566 | 8.245 | 7.112 | 0.999 |
| Spa:Nat - Spa:Hyb | 3.122 | 10.912 | 4.667 | 0.861 |
| SRs:Nat - Spa:Hyb | 0.070 | 7.723 | 7.582 | 1.000 |
| Tun:Nat - Spa:Hyb | 1.7649 | 9.555 | 6.025 | 0.987 |
| Tun:Hyb - SRs:Hyb | 24.241 | 16.008 | 32.474 | < 0.001 |
| Pum:Nat - SRs:Hyb | 21.686 | 13.349 | 30.022 | < 0.001 |
| SRs:Nat - SRs:Hyb | 24.737 | 16.529 | 32.946 | < 0.001 |
| Tun:Nat – SRs:Hyb | 23.043 | 14.707 | 31.380 | < 0.001 |
| Spa:Nat – Tun:Hyb | 2.555 | 10.420 | 5.309 | 0.938 |
| SRs:Nat – Tun:Hyb | 0.496 | 7.232 | 8.225 | 0.999 |
| Tun:Nat – Tun:Hyb | 1.198 | 9.062 | 6.666 | 0.997 |
| SRs:Nat – Spa:Nat | 3.051 | 4.787 | 10.890 | 0.875 |
| Tun:Nat – Spa:Nat | 1.357 | 6.616 | 9.330 | 0.996 |
| Tun:Nat – SRs:Nat | 1.694 | 9.533 | 6.145 | 0.989 |

Supplementary Table 4 showing differences in the number of offspring produced by SR*s* males when introgressed onto 39 isolines across three populations (Spain n=16, Tunisia n=15, UK n=8). Tukey’s post-hoc tests were used to test how the populations differ from each other. The mean number of offspring produced for each isofemale line was calculated using 20-40 replicates. For means per population, see Figure 2b.

| TukeysHSD tests comparing population level off.produced (fig 2b) | | | | |
| --- | --- | --- | --- | --- |
|  | Difference | lower CI | Upper CI | P adjusted |
| Spain-UK | 6.518 | 3.897 | 16.934 | 0.289 |
| Tunisia-UK | 34.293 | 23.762 | 44.824 | < 0.001 |
| Spain-Tunisia | 27.775 | 19.130 | 36.420 | < 0.001 |

Supplementary Table 5 shows the offspring production, offspring sex-ratio and the X-chromosome status of males produced by backcrossing hybrid females carrying one SR*s* and one Spanish X-chromosome to either a Tunisian or a Spanish male. The backcross to a Tunisian male shows rescue of the SR*s* phenotype in 7 males (shown by *). Male types were classified by the offspring sex-ratio: SR*s* if the sex ratio of their offspring was >85% female, non-driving if the sex ratio was 50:50, and unknown if they produced 5 or fewer offspring.

| Male parent | Male No. | Total offspring | % offspring female | X Chromosome ID |
| --- | --- | --- | --- | --- |
| Tunisia | 1* | 54 | 100 | SRs |
|  | 2 | 0 | NA | No ID |
|  | 3* | 18 | 98 | SRs |
|  | 4 | 25 | 44 | Spain |
|  | 5* | 56 | 100 | SRs |
|  | 6 | 0 | NA | No ID |
|  | 7 | 63 | 47 | Spain |
|  | 8* | 59 | 100 | SRs |
|  | 9* | 41 | 95 | SRs |
|  | 10* | 62 | 100 | SRs |
|  | 11 | 71 | 56 | Spain |
|  | 12 | 61 | 46 | Spain |
|  | 13* | 83 | 100 | SRs |
|  | 14 | 62 | 52 | Spain |
|  | 15 | 69 | 47 | Spain |
| Spain | 1 | 52 | 52 | Spain |
|  | 2 | 63 | 55 | Spain |
|  | 3 | 0 | NA | No ID |
|  | 4 | 0 | NA | No ID |
|  | 5 | 1 | NA | No ID |
|  | 6 | 42 | 59 | Spain |
|  | 7 | 0 | NA | No ID |
|  | 8 | 71 | 45 | Spain |
|  | 9 | 45 | 53 | Spain |
|  | 10 | 0 | NA | No ID |
|  | 11 | 24 | 38 | Spain |
|  | 12 | 0 | NA | No ID |
|  | 13 | 1 | NA | No ID |
|  | 14 | 3 | NA | No ID |
|  | 15 | 0 | NA | No ID |
|  | 16 | 1 | NA | No ID |
|  | 17 | 73 | 53 | Spain |
|  | 18 | 0 | NA | No ID |

Supplementary Table 6 Differences between three populations in the proportion of offspring that are female when an SR*s* male was introgressed onto an isoline from that population. Number of isofemale lines used differed across three populations (Spain n=16, Tunisia n=15, UK n=8). Tukey’s post-hoc tests were used to test how the populations differ from each other. The mean proportion of female offspring was calculated for each isofemale line based on 20-40 replicate introgressed males from that isoline. See Figure 5 for the mean sex ratios for each population.

| TukeysHSD tests comparing population level suppression for (fig 5) | | | | |
| --- | --- | --- | --- | --- |
|  | Difference | lower CI | Upper CI | P adjusted |
| Spain-UK | 0.00682 | 0.042 | 0.0556 | 0.937 |
| Tunisia-UK | 0.08567 | 0.135 | 0.0363 | < 0.001 |
| Spain-Tunisia | 0.09250 | 0.133 | 0.0519 | < 0.001 |
