# Supplementary figures and images for: Strong hybrid male incompatibilities impede the spread of a selfish chromosome between populations of a fly
Source: Evol Lett. 2018 May 10;2(3):169–79. doi: 10.1002/evl3.55 (PMC6121854; doi:10.1002/evl3.55)

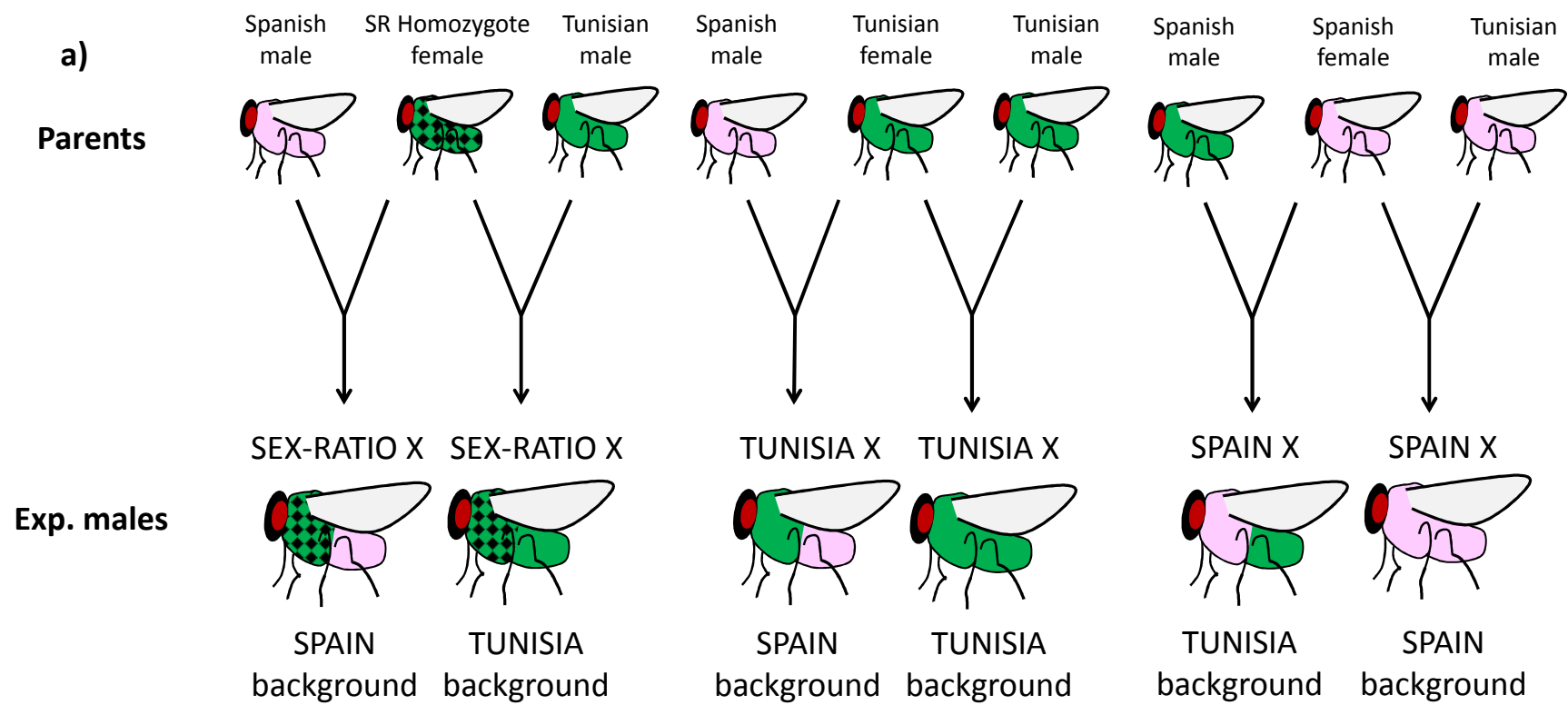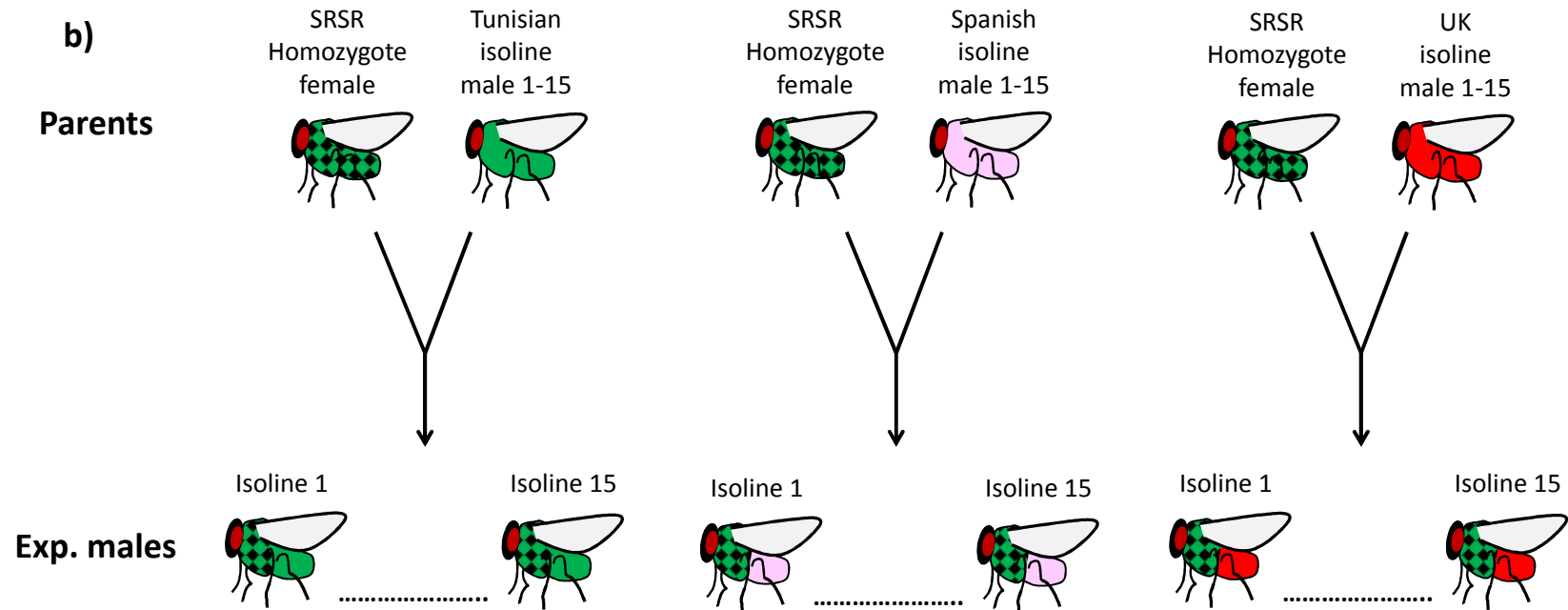

Supplement: Supplementary file 2 — Figure S1. The layout of the crossing schematics for (a) Experiment 1 comparing the fitness of the SRs X chromosome and nondriving X chromosomes from Tunisia and Spain on native and hybrid populations genetic backgrounds; (b) Experiments 2 and 4 comparing the fitness costs of SRs and the levels of suppression of SRs in multiple isofemale lines across three populations. [file EVL3-2-169-s002.pdf]

# KL2 Y chromosome marker

3/9/14

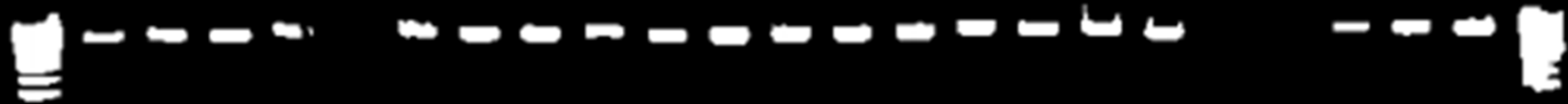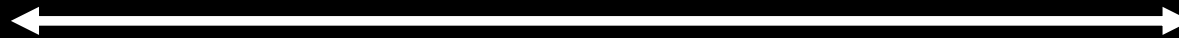

1-18 males from SR fathers

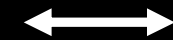

Virgin  
female

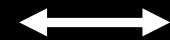

Normal  
male

Supplement: Supplementary file 3 — Figure S2. Gel electrophoresis image showing the amplification of the kl2 gene from the Drosophila subobscura Y chromosome. [file EVL3-2-169-s003.pdf]
